# An easy way to determine bone mineral density and predict pelvic insufficiency fractures in patients treated with radiotherapy for cervical cancer

**DOI:** 10.1007/s00066-020-01690-0

**Published:** 2020-10-06

**Authors:** Drilon Kurrumeli, Markus Oechsner, Bianca Weidenbächer, Christine Brambs, Maximilian Löffler, Stephanie Elisabeth Combs, Kai Borm, Marciana Nona Duma

**Affiliations:** 1 Department of Radiation Oncology, Klinikum rechts der Isar – Technical University of Munich (TUM), Munich, Germany; 2 School of Medicine, Klinikum rechts der Isar – Technical University of Munich (TUM), Munich, Germany; 3 Department of Gynaecology, Klinikum rechts der Isar – Technical University of Munich (TUM), Munich, Germany; 4 Department of Neuroradiology, Klinikum rechts der Isar – Technical University of Munich (TUM), Munich, Germany; 5 Institute of Radiation Medicine (IRM), Department of Radiation Sciences (DRS), Helmholtz Zentrum München (HMGU), Munich, Germany; 6 German Cancer Consortium (DKTK)-Partner Site Munich, Munich, Germany; 7 Department of Radiotherapy and Radiation Oncology, University Hospital Jena, Bachstr. 18, 07743 Jena, Germany

**Keywords:** Gynaecological tumors, Radiation therapy, Magnetic resonance imaging, Cisplatinum

## Abstract

**Purpose:**

The aim of this study was to investigate whether bone mineral density (BMD) as measured in planning computed tomographies (CTs) by a new method is a risk factor for pelvic insufficiency fractures (PIF) after radio(chemo)therapy (R(C)T) for cervical cancer.

**Methods:**

62 patients with cervical cancer who received definitive or adjuvant radio(chemo)therapy between 2013 and 2017 were reviewed. The PIF were detected on follow-up magntic resonance imaging (MRI). The MRI of the PIF patients was registered to the planning CT and the PIF contoured. On the contralateral side of the fracture, a mirrored structure of the fracture was generated (mPIF). For the whole sacral bone, three lumbar vertebrae, the first and second sacral vertebrae, and the PIF, we analyzed the BMD (mg/cm^3^), V50Gy, Dmean, and Dmax.

**Results:**

Out of 62 patients, 6 (9.7%) had a fracture. Two out of the 6 patients had a bilateral fracture with only one of them being symptomatic. PIF patients showed a significantly lower BMD in the sacral and the lumbar vertebrae (*p* < 0.05). The BMD of the contoured PIF, however, when comparing to the mPIF, did not reach significance (*p* < 0.49). The difference of the V50Gy of the sacrum in the PIF group compared to the other (OTH) patients, i.e. those without PIF, did not reach significance.

**Conclusion:**

The dose does not seem to have a relevant impact on the incidence of PIF in our patients. One of the predisposing factors for developing PIF after radiotherapy seems to be the low BMD. We presented an easy method to assess the BMD in planning CTs.

## Introduction

Definitive or adjuvant radio(chemo)therapy have both become an essential component in the treatment of cervical cancer, especially in locally advanced cervical cancer (LACC), by reducing the recurrence of the disease and improving the overall survival rate for these patients [1–5]. Definitive treatment consists of concomitant radiochemotherapy and brachytherapy or external beam radiation therapy (EBRT) alone and brachytherapy. EBRT can be applied as concomitant radiochemotherapy with a total dose of 45–50 Gy (1.8 Gy per fraction) and single-agent radio-sensitizing chemotherapy, preferably cisplatin (weekly 40 mg/m^2^). Boost treatment for involved lymph nodes may be applied as simultaneous integrated boost (SIB) within the EBRT treatment or as sequential boost (SB). In large tumors brachytherapy should be delivered within 1–2 weeks towards the end of or after radiochemotherapy. In tumors of limited size brachytherapy may start earlier during radiochemotherapy. The aim should be to reach a total EBRT + brachytherapy dose of ≥85–90 Gy. Additional brachytherapy as part of the adjuvant treatment should only be considered if a well-defined limited area is at high risk of local recurrence (i.e., vagina, parametrium) [4, 6]. However, through these advances in treatment, the late effects of pelvic radiation have drawn more attention. Pelvic insufficiency fractures (PIF) are fractures caused by normal or physiological stress on bone with demineralization and decreased elastic resistance. The lateral mass of the sacrum is the most commonly affected site because of its weight-bearing function [7]. Several studies investigating the incidence of PIF showed a wide incidence range from 1.7 to 89% [8–11], which leads to the belief that PIF are a rather common postradiation complication and not as rare as previously thought. Risk factors for pelvic fractures after radiotherapy for cervical cancer are yet to be fully understood. Oh et al. [12] found that age ≥55 years and body weight <55 kg were significant predisposing factors for developing PIF. In the Schmeler et al. [13] study, postmenopausal status was also a significant risk factor. However, very few studies have been able to evaluate bone mineral density (BMD) as a risk factor for the occurrence of PIF. Uezono et al. [14] was one of the few studies to associate lower CT density with the development of PIF. Osteoporosis, a disease in which bone weakening increases the risk of fracture, plays an important role in the development of PIF. The T‑score is an important parameter when screening for osteoporosis. It is the BMD compared to the mean value of a healthy young adult. Osteoporosis is defined as a T-score of −2.5 or lower, meaning a BMD two and a half standard deviations below the mean of a young healthy adult reference. The BMD can predict osteoporotic vertebral fractures [15]. Established methods of assessing bone mineral density are associated with additional radiation exposure to the patient.

There are no data on BMD measurements based on the planning CTs for radiation therapy up to date.

The aim of this study was to investigate whether bone mineral density as measured in planning CTs is a risk factor for pelvic insufficiency fractures (PIF) after radio(chemo)therapy (R(C)T) for cervical cancer.

## Materials and methods

### Patients

Medical records of 62 patients with cervical cancer who received treatment between 2013 and 2017 at the Department of Radiation Oncology of the Klinikum Rechts der Isar, Munich, Germany, were reviewed. The median age was 55 years (32–81 years). Patients were treated with EBRT alone or EBRT and concurrent chemotherapy. 33 patients were treated with definitive radiochemotherapy, 22 with adjuvant radiochemotherapy, 5 with definitive radiotherapy, and 2 patients were treated with adjuvant radiotherapy. 31 patients also received additional high-dose-rate intracavitary brachytherapy (HDR-ICBT). 43 patients received an EBRT boost to the primary tumor (PT) and/or the lymph nodes (LN), with 34 of them receiving a simultaneous integrated boost (SIB), 13 a sequential boost (SB), and amongst them, 4 patients received both boost techniques (first SIB, then SB). The median dose to the planning target volume (PTV) was 50.4 Gy (36–50.4 Gy). The median EBRT boost dose to the PT was 56 Gy (range 42–70 Gy) and to the LN 58.8 Gy (range 52–61.8 Gy). The median dose delivered through HDR-ICBT was 28 Gy (range 5–28 Gy). Out of the 55 patients who underwent concomitant chemotherapy, 52 patients received platin-based chemotherapy (cisplatin or carboplatin), while 3 patients were treated with vinorelbine. 7 patients did not undergo chemotherapy because of either medical contraindication or the patient’s refusal.

The UICC stages of our patients are described in Table 1.

**Table 1:** TNM-stage and grade distribution

T	–	T1	T2	T3	T4	Tx
*n *(%)	–	19 (30.6)	27 (43.5)	11 (17.7)	4 (6.5)	1 (1.6)
N	N0	N1	–	–	–	–
*n *(%)	17 (27.4)	45 (72.6)	–	–	–	–
M	M0	M1	–	–	–	Mx
*n *(%)	40 (64.5)	19 (30.6)	–	–	–	3 (4.8)
Grading	–	G1	G2	G3	–	Gx
*n *(%)	–	1 (1.6)	32 (51.6)	28 (45.2)	–	1 (1.6)

### Imaging analysis

All patients underwent follow-up magnetic resonance imaging (MRI). The PIF were detected in the follow-up MRIs by experienced radiologists. In the MRI findings the PIF were defined as hypointense signal alterations on T1- or hyperintense signal alterations on T2-weighted images. We registered the MRI of the patients in the PIF group into the planning computed tomography scan (CT) and re-contoured the PIF region on the planning CT with MRI guidance using the ARIA oncology information system (Varian Medical Systems, Palo Alto, CA, USA). Then, on the contralateral side of the fracture, a mirrored structure of the fracture (mPIF) was generated. We also manually contoured the sacrum for all patients.

We analyzed dose–volume histogram (DVH) parameters such as the V30, V40, and V50 of the sacrum and the PIF for each patient. We also analyzed the D50%, which gives us the dose that 50% of the volume of the sacrum/PIF received, as well as the Dmean and the Dmax, which provide information about the mean/maximum dose these structures were irradiated with, respectively.

We also analyzed the BMD of three lumbar vertebrae, as well as of the first and second sacral vertebrae for each patient, and compared the results of the patients with PIF to those without. For the lumbar vertebrae we analyzed the first to the third lumbar body in the planning CT of patients; where the first or the second lumbar vertebra wasn’t completely depicted, we analyzed the second to the fourth and the third to the fifth, respectively.

**Fig. 1:**
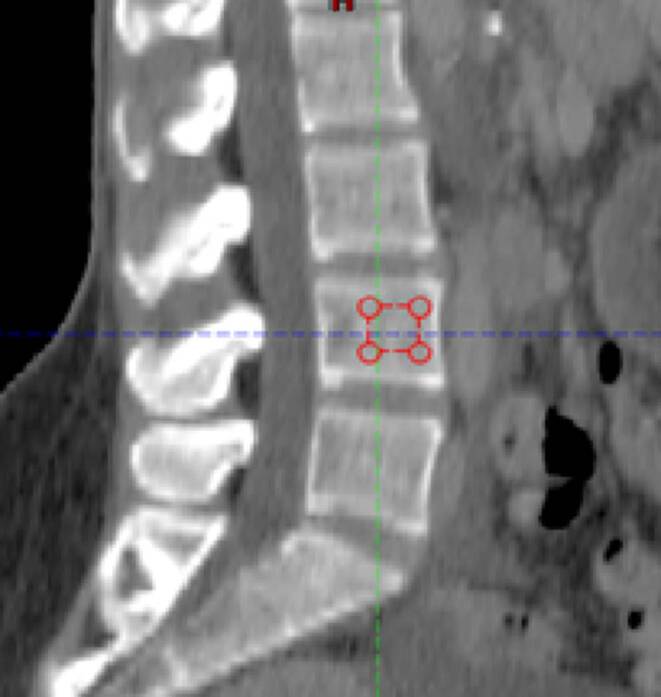
Region of interest (ROI) placement for the measurement of CT density (in HU) in the planning CT. In the sagittal plane of the planning CT, a square ROI (*red*) half the height of the lumbar vertebra was placed in the ventral half of the trabecular compartment of the fourth lumbar body by tilting the CT image in order for the trabecular compartment to be parallel to the sides of the square ROI and was subsequently used to show mean attenuation values of the trabecular bone (in HU)

For this, similar to the Schwaiger et al. publication [15], in the sagittal plane of the planning CT for each patient, we placed a square region of interest (ROI) half the height of the lumbar vertebrae. The square was placed in the ventral half of the trabecular compartment of the vertebrae by tilting the CT image in order for the trabecular compartment to be in a parallel position to the sides of the square ROI and was subsequently used to show mean attenuation values of the trabecular bone in Hounsfield units (HU; Fig. 1).

Then we used a multidetector row CT (MDCT)-to-quantitative CT (qCT) equation to convert the obtained HU values from planning CTs into BMD (mg/cm^3^).

### Statistical analysis

The statistical analyses were performed with IBM SPSS Statistics 25.0 (IBM, Armonk, NY, USA). Correlation between the BMD and PIF, as well as between the dose–volume histogram parameters and PIF, were analyzed by *t*-test. A value of *P* < 0.05 was considered to be statistically significant.

## Results

**Fig. 2:**
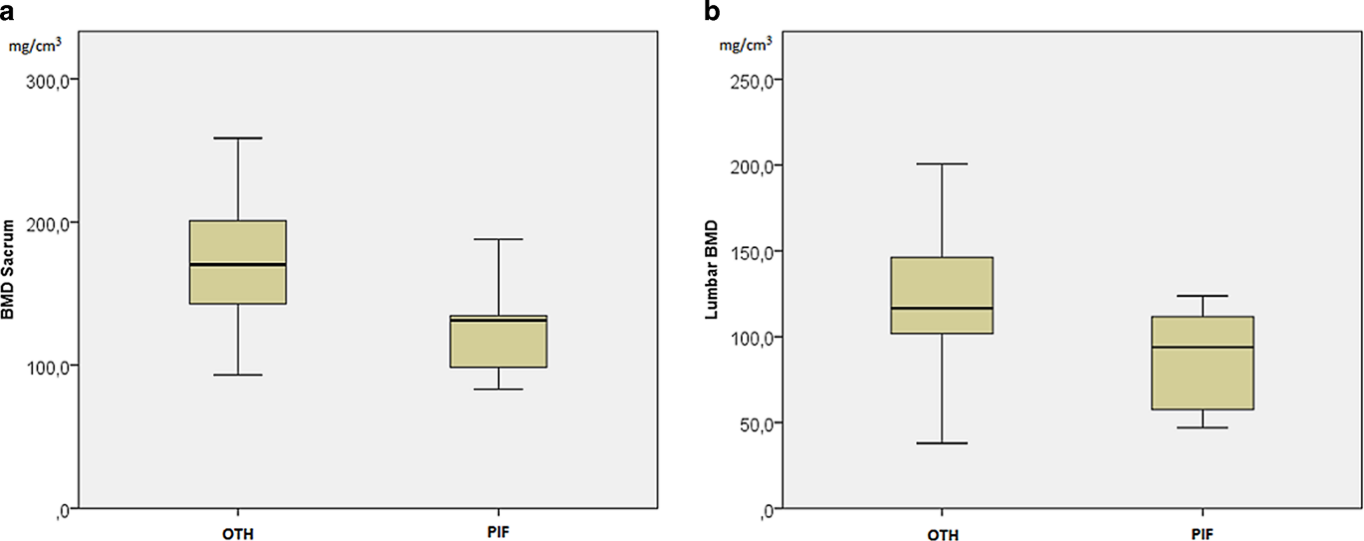
Boxplot depiction of the bone mineral density (*BMD*) of the sacrum (**a**) and lumbar vertebrae (**b**) in patients with a pelvic insufficiency fracture (*PIF*) and in those without (*OTH*)

Out of 62 patients, 6 (9.7%) had a fracture. All of them were detected in follow-up MRIs as PIFs and all of them were located in the lateral mass of the sacral bone. The median imaging follow-up was 16 months (range 1.5–53 months). Two out of those 6 patients had a bilateral fracture, with only one of them being symptomatic. The median age in the PIF group was significantly higher (*p* < 0.01), with 62 years compared to 53 years in the other patients (OTH), i.e., without PIF. The median time between radiotherapy and the detected fracture was 6.5 months (range 5–9.7). The BMD of the sacral bone was significantly lower (*p* < 0.03) in the PIF group, with 127.8 mg/cm^3^ compared to 173.1 mg/cm^3^ in the OTH group. There was a significant (*p* < 0.05) difference of the mean BMD of the lumbar vertebrae in the PIF group (87.9 mg/cm^3^) and the OTH group (121.4 mg/cm^3^; Fig. 2). However, the differences in the PIF compared to the mPIF and the first and second sacral bodies in the PIF group compared to the ones in the OTH group were both nonsignificant (*p* < 0.49 and *p* < 0.15, respectively). The BMD association with PIF is shown in Table 2.

**Table 2:** Bone mineral density association with pelvic insufficiency fractures

	PIF group (*n* = 6)	OTH (*n* = 56)	*P*-value
BMD_sacrum_, mg/cm^3^	127.8	173.1	0.03
BMD_lumbar_, mg/cm^3^	87.9	121.4	0.05
BMD_S1,2_, mg/cm^3^	79.3	107.8	0.15
BMD_PIF/mPIF_, mg/cm^3^	70.4/84.2	–	0.49

*PIF* pelvic insufficiency fractures, *mPIF* mirrored PIF (the same structure as PIF mirrored on the healthy side of the sacrum), *OTH* other patients, i.e., patients without a fracture, *BMD* bone mineral density

The Dmean/Dmax of the sacrum in the PIF group were 39.5 Gy/55.1 Gy and in OTH 39.7 Gy/53.9 Gy, respectively (with no significant difference between groups). The Dmean/Dmax of the PIF, although higher than in the mPIF, did not reach significance (*p* < 0.20 and *p* < 0.48, respectively). We performed dosimetric analyses of EBRT on all our patients. The differences in D50% of the sacrum/PIF between our study groups were both nonsignificant (*p* < 0.89 and *p* < 0.31, respectively). In our study group we found a significant difference in the V30, V40, and V50. The full list of analyzed DVH parameters is shown in Table 3.

**Table 3:** Dose-volume histogram parameter association with PIF

	PIF group (*n* = 6)	OTH (*n* = 56)	*P*-value
Dmean_sacrum_, Gy	39.5	39.7	0.87
Dmean_PIF/mPIF_, Gy	41.3/35	–	0.20
Dmax_sacrum_, Gy	55.1	53.9	0.44
Dmax_PIF/mPIF_, Gy	52.5/51.7	–	0.48
D50%_sacrum_, Gy	41.2	40.9	0.89
D50% _PIF/mPIF_, Gy	42.5/36	–	0.31
V30Gy_sacrum_, %	82.5	83.3	0.91
V30Gy_PIF/mPIF_, %	83.7/66.6	–	0.36
V40Gy_sacrum_, %	52.7	54.7	0.78
V40Gy_PIF/mPIF_, %	60.6/37.6	–	0.24
V50Gy_sacrum_, %	16.2	13.7	0.61
V50Gy_PIF/mPIF_, %	25.2/7.8	–	0.11

*PIF* pelvic insufficiency fractures, *mPIF* mirrored PIF (the same structure as PIF mirrored on the healthy side of the sacrum), *OTH* Other patients, i.e. patients without a fracture, *BMD* bone mineral density, V30/40/50 Gy- the relative volume that received 30/40/50 Gy, *Dmax* Maximum dose, *Dmean* Mean

## Discussion

In our study we found that approximately 10% of cervical cancer patients will suffer a pelvic insufficiency fracture (PIF).

This study strengthens the assumption of recent studies that PIFs are not as rare a complication after radiotherapy as previously thought and might have been underestimated in gynecological patients. Several studies investigating the incidence of PIF showed a wide range of incidence from 1.7 to 89% [8–11], with the more recent ones [10–14] describing higher incidence rates than the earlier ones. Blomlie et al. [8] was an exception, as they described an incidence of 89% back in 1996. The reason for the overall higher incidence rate in recent studies may be the attention this postradiation complication has received in recent years, as well as the improvement of follow-ups (i.e., the frequency and timing of the scans). Several studies focused on risk factors for PIF.

Gondi et al. [16] investigated severe late toxicities after either radiotherapy or radiochemotherapy for LACC and compared these two groups. They found a significant relation between PIF and patients in the radiochemotherapy group. Various other studies investigating the same phenomenon were not able to show similar significant relations of PIF and patients receiving concomitant chemotherapy [11, 13, 14]. The same was true for our study, as we were also unable to find a significant relation (*p* < 0.74).

Ramlov et al. [11] found that PIFs occurred mainly in patients aged >50 years, a group of patients who are predominantly postmenopausal, where lower bone density plays an important role. Another study found that the incidence of PIF after radiotherapy is low (4.4%), but is significantly higher in patients aged >50 years and in postmenopausal patients [17]. However, in this study, the osteoporosis status was available for only 9% of their patients, and the pretreatment bone density was not evaluated. The median age in patients with PIF was significantly higher (*p* < 0.01) in our study (62 years compared to 53 years).

Several studies have investigated the relation between the dose delivered to the sacrum and PIF. Uezono et al. [14] did not find a significant relation between DVH parameters and the risk of PIF. Although not in the whole patient cohort, Ramlov et al. [11] did find a statistically significant correlation in the group of patients aged >50 years, according to which a decrease in D50%_sacrum_ dose from 40 to 35 Gy results in an absolute decrease in risk of fracture by 23%, from 45 to 22%. Other DVH parameters, such as the V55Gy, did not reach significance. In our study, we did not observe a statistically significant relation of DVH parameters and the incidence of PIF.

In a 2020 study, Sapienza et al. [18] evaluated the incidence of PIF in a meta-analysis and meta-regression. Twenty-one studies with a total of 3929 patients were included. The overall PIF incidence was 14%. The sacrum and the sacroiliac joint were the most common sites of PIF. In this study, the use of intensity-modulated radiation therapy, which is mostly used in the modern era, appeared to come with a lower risk for developing PIF. The median time between the end of radiotherapy and the occurrence of the insufficiency fracture was 7.1 to 19 months. As called for by the authors of this study, posttreatment bone surveillance is essential in these patients, as a large group of patients are asymptomatic by the time the PIF is diagnosed. This is very similar to our data, with approximately 10% PIF and low symptom burden due to PIF.

Very few studies have been able to evaluate BMD as a risk factor for PIF. BMD is usually assessed using dual-energy X‑ray absorptiometry (DXA) or the qCT. DXA uses two X‑ray beams that are directed at the patient’s bones. After the absorption of the soft tissue is deducted, the BMD can be determined from the absorption of each beam by bone. The qCT makes use of a standard X‑ray CT scanner with a calibration standard. In this way, it converts the HU obtained from the CT image into BMD. While we know the usual methods of assessment of BMD like DXA and qCT to be well established and mostly very accurate, there is some variability in qCT validity which is highly dependent on the technician performing the analysis [15].

Schwaiger et al. [15] assessed the BMD of 106 vertebral bodies in 38 patients in qCT as a standard of reference and in sagittal reformations derived from MDCT. MDCT-to-qCT conversion equations were calculated and then applied to baseline MDCTs for another 62 patients. After a mean follow-up of 15 ± 6 months, patients were re-assessed for incidental fractures and screw loosening after spondylodesis. Patients who developed incidental fractures during follow-up showed significantly lower baseline MDCT-BMD values than patients without incidental fractures. Also, patients with spondylodesis and signs of screw loosening had significantly lower MDCT-BMD values than patients without screw loosening. This longitudinal study showed that converted BMD values can differentiate patients with or without osteoporotic fractures at baseline, as well as predicting incidental fractures and screw loosening in patients with spondylodesis during follow-up.

Uezono et al.[14] measured pretreatment CT density (in HU) by selecting three different axial images showing visually the lowest bone marrow density on bone windows in the right and the left sacrum. Then, circular regions of interest (ROI) on each side of the sacrum were generated to measure the CT density of bone and bone marrow on each of the three different axial images. For the L5 vertebra they selected the axial image that appeared the most homogenous. Each of three values (L5 vertebra, right and left sacrum) and the mean density of the three were analyzed and showed a significant correlation between low CT density and the occurrence of PIF. They evaluated the imaging records of 99 patients who were treated at their institution between 2003 and 2009, but could only measure the pretreatment CT density of 59 (59.6%) of those 99 patients, meaning a large number of the patients’ HU were not available. Imaging data of those 59 patients were acquired in or after 2006 and were therefore obtainable through their specific imaging viewer (Synapse) with the option of CT densitometry. However, data of the other 40 (40.4%) patients acquired before 2006 were not obtainable through the imaging viewer.

In our study we were able to evaluate the BMD of every patient from the collected data from existing CT scans and converted the HU into mg/cm^3^ with the help of an MDCT-to-qCT equation, consequently making the need of a qCT, which brings along additional radiation exposure, redundant.

Based on our study, a lower BMD (measured by an MDCT-to-qCT equation) is a significant predisposing factor for developing PIF after radiotherapy.

Osteoporosis is known to be one of the most common causes of insufficiency fractures [19]. One strength of our study is that BMD assessment by an MDCT-to-qCT equation—as previously shown by Schwaiger et al. [15]—is predictive for bone health. The new approach presented herein (a BMD measurement in the planning CT) enhances the radiation oncologist’s armamentarium in predicting the individual toxicity risk of a patient. This information can be used in clinical practice and should be used in future prospective studies to predict osteoporosis and osteoporotic fractures after radiotherapy and implement early countermeasures.

A limitation of our study is the fact that this was a single-institution, retrospective study with a rather small number of patients. Therefore, the likelihood of finding statistically significant correlations was low. In addition, follow-up imaging studies were not performed at specified intervals and were mainly targeted to the primary disease, which may have led to missed asymptomatic fractures outside the imaging interval. Further, no osteoporosis screening was performed. In our study group, none of the patients had a diagnosed osteoporosis as a pre-existing disease. Since most of our patients were still relatively young (with a median age of 55) and none of them had had any symptoms or complications that may have led to the diagnosis of osteoporosis, we did not find it necessary or practicable to routinely screen every patient.

## Conclusion

PIFs are a common complication after radiotherapy in patients with cervical cancer. In our study they were detected these in 9.7% of the patients. The dose did not seem to have a significant impact on the incidence of PIF in our group of patients. Predisposing factors for developing postradiation PIF seem to be older age and a lower BMD. Herein, we report an easy way to determine BMD in the planning CT for radiotherapy without additional radiation exposure for the patient.

## References

[1] Morris M (1999). Pelvic radiation with concurrent chemotherapy compared with pelvic and para-aortic radiation for high-risk cervical cancer. N Engl J Med.

[2] Keys HM (1999). Cisplatin, radiation, and adjuvant hysterectomy compared with radiation and adjuvant hysterectomy for bulky stage IB cervical carcinoma. N Engl J Med.

[3] Rotman M (1995). Prophylactic extended-field irradiation of para-aortic lymph nodes in stages IIB and bulky IB and IIA cervical carcinomas. Ten-year treatment results of RTOG 79-20. JAMA.

[4] Sedlis A (1999). A randomized trial of pelvic radiation therapy versus no further therapy in selected patients with stage IB carcinoma of the cervix after radical hysterectomy and pelvic lymphadenectomy: a gynecologic oncology group study. Gynecol Oncol.

[5] Rose PG (1999). Concurrent cisplatin-based radiotherapy and chemotherapy for locally advanced cervical cancer. N Engl J Med.

[6] Leitlinienprogramm Onkologie (2014) S3-Leitlinie Diagnostik und Therapie Nachsorge der Patientin mit Zervixkarzinom. https://www.leitlinienprogramm-onkologie.de/fileadmin/user_upload/Downloads/Leitlinien/Zervixkarzinom/LL_Zervixkarzinom_Langversion_1.0.pdf. Accessed 2019

[7] Cooper KL, Beabout JW, Swee RG (1985). Insufficiency fractures of the sacrum. Radiology.

[8] Blomlie V (1996). Incidence of radiation-induced insufficiency fractures of the female pelvis: evaluation with MR imaging. AJR Am J Roentgenol.

[9] Huh SJ (2002). Pelvic insufficiency fracture after pelvic irradiation in uterine cervix cancer. Gynecol Oncol.

[10] Tokumaru S (2012). Insufficiency fractures after pelvic radiation therapy for uterine cervical cancer: an analysis of subjects in a prospective multi-institutional trial, and cooperative study of the Japan radiation oncology group (JAROG) and Japanese radiation oncology study group (JROSG). Int J Radiat Oncol Biol Phys.

[11] Ramlov A (2017). Risk factors for pelvic insufficiency fractures in locally advanced cervical cancer following intensity modulated radiation therapy. Int J Radiat Oncol Biol Phys.

[12] Oh D (2008). Pelvic insufficiency fracture after pelvic radiotherapy for cervical cancer: analysis of risk factors. Int J Radiat Oncol Biol Phys.

[13] Schmeler KM (2010). Pelvic fractures after radiotherapy for cervical cancer: implications for survivors. Cancer.

[14] Uezono H (2013). Pelvic insufficiency fracture after definitive radiotherapy for uterine cervical cancer: retrospective analysis of risk factors. J Radiat Res.

[15] Schwaiger BJ (2014). Bone mineral density values derived from routine lumbar spine multidetector row CT predict osteoporotic vertebral fractures and screw loosening. AJNR Am J Neuroradiol.

[16] Gondi V (2012). Severe late toxicities following concomitant chemoradiotherapy compared to radiotherapy alone in cervical cancer: an inter-era analysis. Int J Radiat Oncol Biol Phys.

[17] Bazire L (2017). Pelvic insufficiency fracture (PIF) incidence in patients treated with intensity-modulated radiation therapy (IMRT) for gynaecological or anal cancer: single-institution experience and review of the literature. Br J Radiol.

[18] Sapienza LG (2020). Pelvic insufficiency fractures after external beam radiation therapy for gynecologic cancers: a meta-analysis and meta-regression of 3929 patients. Int J Radiat Oncol Biol Phys.

[19] Krestan C, Hojreh A (2009). Imaging of insufficiency fractures. Eur J Radiol.

